# The gasdermins: a pore-forming protein family expressed in the epidermis

**DOI:** 10.3389/fimmu.2023.1254150

**Published:** 2023-09-12

**Authors:** Marta Slaufova, Tugay Karakaya, Michela Di Filippo, Paulina Hennig, Hans-Dietmar Beer

**Affiliations:** ^1^Department of Dermatology, University Hospital Zurich, Zurich, Switzerland; ^2^Faculty of Medicine, University of Zurich, Zurich, Switzerland

**Keywords:** gasdermin (GSDM), pyroptosis, inflammasome, skin, interleukin - 1

## Abstract

Gasdermins comprise a family of pore-forming proteins, which play critical roles in (auto)inflammatory diseases and cancer. They are expressed as self-inhibited precursor proteins consisting of an aminoterminal cytotoxic effector domain (NT-GSDM) and a carboxyterminal inhibitor domain (GSDM-CT) separated by an unstructured linker region. Proteolytic processing in the linker region liberates NT-GSDM, which translocates to membranes, forms oligomers, and induces membrane permeabilization, which can disturb the cellular equilibrium that can lead to cell death. Gasdermin activation and pore formation are associated with inflammation, particularly when induced by the inflammatory protease caspase-1 upon inflammasome activation. These gasdermin pores allow the release of the pro-inflammatory cytokines interleukin(IL)-1β and IL-18 and induce a lytic type of cell death, termed pyroptosis that supports inflammation, immunity, and tissue repair. However, even at the cellular level, the consequences of gasdermin activation are diverse and range from induction of programmed cell death - pyroptosis or apoptosis - to poorly characterized protective mechanisms. The specific effects of gasdermin activation can vary between species, cell types, the membrane that is being permeabilized (plasma membrane, mitochondrial membrane, etc.), and the overall biological state of the local tissue/cells. In epithelia, gasdermins seem to play crucial roles. Keratinocytes represent the main cell type of the epidermis, which is the outermost skin layer with an essential barrier function. Compared to other tissues, keratinocytes express all members of the gasdermin family, in part in a differentiation-specific manner. That raises questions regarding the specific roles of individual GSDM family members in the skin, the mechanisms and consequences of their activation, and the potential crosstalk between them. In this review, we summarize the current knowledge about gasdermins with a focus on keratinocytes and the skin and discuss the possible roles of the different family members in immunity and disease.

## Introduction

The skin represents the outer barrier of the human body and is in permanent contact with the environment (1). It limits water loss from the body and protects from physical stressors, such as UV radiation, and pathogens (2). The epidermis, the outermost layer of the skin, is a constantly renewing stratified squamous epithelium, consisting almost exclusively of a single cell type, termed keratinocytes, in different stages of differentiation (1). In the basal layer, keratinocyte stem cells divide, relocate upwards, and terminally differentiate, giving rise to distinct layers of cells characterized by a specific pattern of expressed genes. The terminal differentiation of keratinocytes shares similarities with programmed cell death pathways, such as the requirement for the activation of caspases (3). It culminates in the formation of corneocytes (4, 5), anucleated and dead keratinocytes, which form a cornified envelope, the outermost layer of the epidermis indispensable for the skin’s barrier function (6). Moreover, keratinocytes are also actively involved in anti-bacterial and anti-viral defense and induce immune responses by expressing and secreting anti-microbial peptides and pro-inflammatory cytokines in a constitutive or inducible manner (7, 8). Therefore, the epidermis and keratinocytes have important functions in immunity (2, 9).

The name gasdermin (GSDM) originates from the identification of a murine gene (now termed *GsdmA3*), which is mainly expressed in the gastrointestinal tract and the skin (dermis) (10). It was shown that mutations of the *GsdmA3* gene induced by N-ethyl-N-nitrosourea cause alopecia in mice (11, 12). Later, it turned out that mice express several Gsdms (13), which have homology to the human deafness autosomal dominant nonsyndromic sensorineural 5 (DFNA5) protein (now GSDME). Due to several gene duplications and deletions, the GSDM family consists of six members in humans, with *GSDME* as the most ancient one and linked to autosomal dominant nonsyndromic hearing loss (14, 15). In contrast, mice express ten different Gsdms (16).

Gasdermins induce a lytic type of cell death termed pyroptosis (17, 18). Although initially termed apoptosis, the process of pyroptosis was first described in macrophages infected by *Shigella flexneri* (19). Later, other bacteria were identified to be able to induce a lytic type of cell death regulated by the cysteine protease caspase-1 (20, 21), such as *Salmonella* (22, 23), *Shigella flexneri* (24), *Listeria* (25), *Pseudomonas aeruginosa* (26), *Legionella pneumophilia* (27), and *Yersinia* (28).

Caspase-1 belongs to a family of aspartate-specific cysteine proteases, which have essential roles in regulated cell death pathways (29). Caspase-1, caspase-4, and caspase-5 (and caspase-11, which is the murine homologue of human caspase-4 and -5) are termed “inflammatory” caspases as their activation is associated with the induction of inflammation (30). Caspase-1, supported by caspase-4/-5 (or -11 in mice), activates the pro-inflammatory cytokines IL-1β and IL-18, which are expressed as biologically inactive precursor proteins (31). Both cytokines are members of the IL-1 family of inflammatory cytokines and play fundamental roles in the induction and regulation of inflammatory responses and immunity (32, 33).

Caspase-1 is initially expressed as an enzymatically inactive precursor protein and proteolytically self-activates upon activation and assembly of different protein complexes, termed inflammasomes (34–36). Inflammasomes consist of a sensor protein, such as NLRP1 (nucleotide-binding domain, leucine-rich-containing family, pyrin domain-containing-1), NLRP3 or AIM2 (absent in melanoma 2), the adaptor protein ASC (apoptosis-associated speck-like protein containing a caspase recruitment domain), and the protease caspase-1. Sensing of different exogenous pathogen-associated molecular patterns (PAMPs) or endogenous damage-associated molecular patterns (DAMPs) induces oligomerization of the sensor, recruitment of ASC with the subsequent formation of large ASC polymers (termed ASC specks), and finally, proteolytic self-activation of pro-caspase-1. Then, caspase-1 cleaves and thereby activates proIL-1β and -18 (and GSDMD, see below), which upon secretion, induce inflammation. This inflammatory response is required for repair processes and immunity (37, 38). However, chronic inflammasome activation, particularly the NLRP3 inflammasome, underlies the pathology of numerous (auto)inflammatory diseases and contributes to cancer development (36, 39–42).

NLRP1 is considered the central inflammasome sensor in human skin and is expressed by keratinocytes (43, 44). Human NLRP1 is activated by UVB radiation (45, 46), underlying sunburn, and p38 activation (47–49), as well as by viral 3C proteases (50), double-stranded RNA (51), and by talabostat, an anticancer drug and dipeptidyl peptidase 8/9 inhibitor (52). Single nucleotide polymorphisms (SNPs) of *NLRP1* are associated with (auto)inflammatory diseases affecting mainly the skin, such as vitiligo (43, 44, 53). Gain-of-function mutations of *NLRP1* cause inflammatory skin syndromes, which predispose patients to the development of squamous cell carcinoma, a type of keratinocyte-derived skin cancer (54, 55). The NLRP1 pathway is poorly conserved in mice (56). Although sunburn in mice is caspase-1- and IL-1-dependent, this process is not regulated by the expression of these proteins by keratinocytes but most likely by other immune cells in the skin (57).

IL-1β and IL-18 lack a signal peptide for secretion by the canonical endoplasmic reticulum/Golgi-dependent pathway and are released by several mechanisms and pathways, collectively termed unconventional protein secretion (58–60). Secretion of these cytokines is regulated by caspase-1 activity upon inflammasome activation (61). This is mediated by the aminoterminal GSDMD fragment (NT-GSDMD), generated by caspase-1 upon cleavage at aspartate 275 (62–64). NT-GSDMD forms pores in the plasma membrane that allow the release of IL-1β and -18 (65–68). Furthermore, GSDMD is also activated by caspase-4/-5/-11 upon noncanonical inflammasome activation induced by LPS. LPS binds and thereby activates caspase-4/-5/-11 directly. Although LPS doesn’t activate caspase-1 directly, caspase-4/-5/-11-induced pyroptosis leads to secondary NLRP3 activation and downstream cleavage of caspase-1, proIL-1β, and proIL-18 (63, 69, 70).

## The gasdermin family: an overview

The human gasdermin family consists of six members (GSDMA to GSDMF), whereas mice express ten different Gsdms. Among them are three GsdmA and four GsdmC (**Table 1**) (16). GSDMB is expressed only in humans (17). Humans and mice express GSDMF/GsdmF (also termed pejvakin/PJVK or DFNB59), which shares sequence and functional similarity with GSDME because mutations of both of these genes cause hearing loss in humans (14, 71, 72). However, GSDMF is structurally different from the other GSDM family members, and it is not known whether it can form membrane pores (17). Therefore, although it belongs to the GSDM family, it is not discussed in this review. Human and murine gasdermin proteins share the same structural organization into a conserved aminoterminal polypeptide (NT-GSDM) and a less conserved carboxyterminal part (GSDM-CT) with variable length (which is missing in GSDMF) separated by an unstructured linker region (17, 73).

**Table 1:** GSDM genes and their chromosomal localization (13, 16, 17).

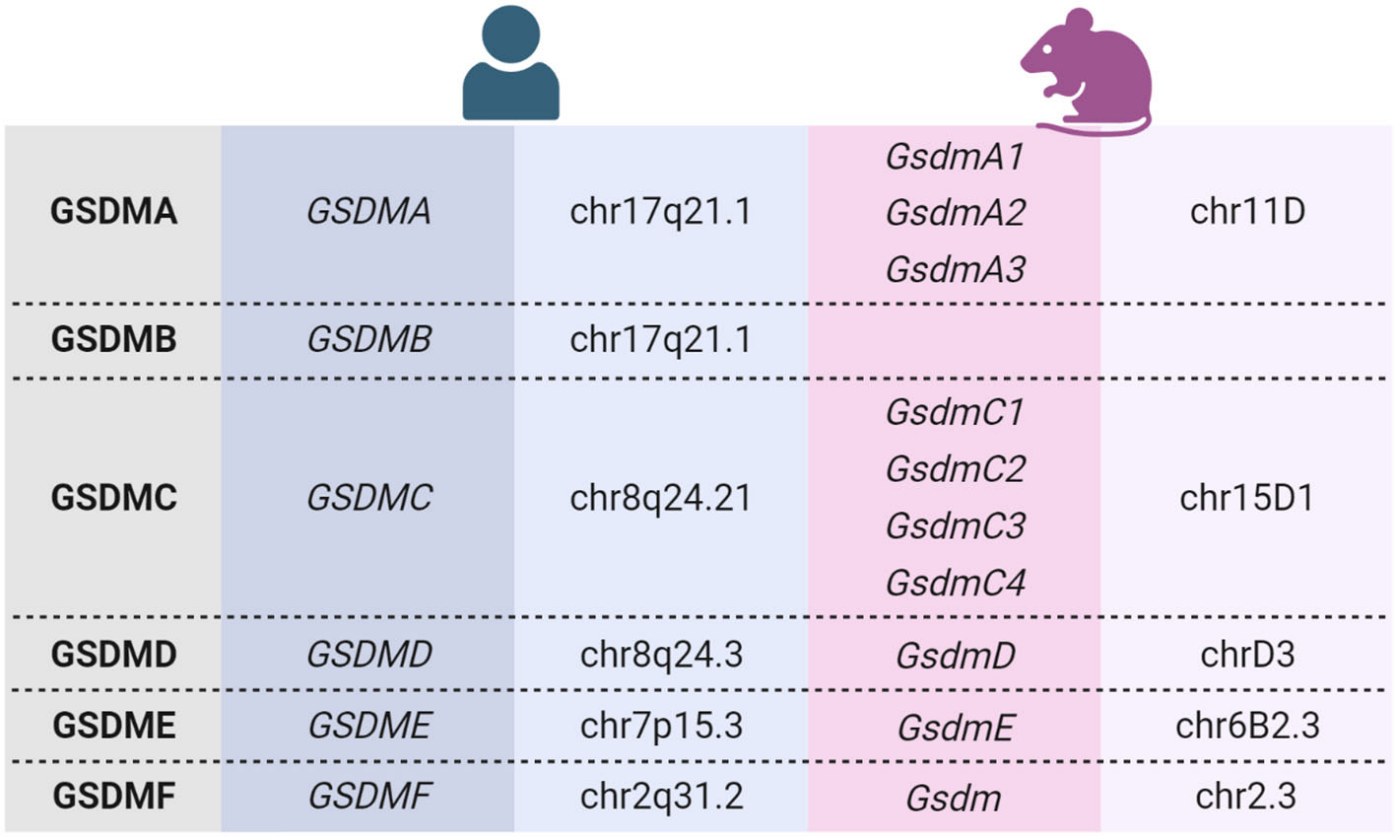

Mutations in GsdmA3-CT, particularly in the stretch of amino acids 343-348, cause alopecia in mice (11, 74). However, mice lacking GsdmA3 expression do not show a spontaneous phenotype (75), whereas GsdmA3 overexpression causes epidermal hyperplasia and skin inflammation (75, 76). Overexpression of GsdmA3 in cultured cells suggested that GsdmA3-CT inhibits the activity of NT-GsdmA3 (74, 77), which was confirmed by cryo-EM (78). Mutations of human GSDMA, GSDMC, GSDMD, and GSDME in the region of GSDM-CT, where mutations induce alopecia in mice, also cause their activation and pyroptosis (65). Therefore, NT-GSDM is the pore-forming effector domain inhibited by GSDM-CT (79).

Wild type GSDMs are activated by proteolytic processing in the linker region, thereby releasing pore-forming NT-GSDM, which inserts into membranes and forms pores upon oligomerization (73). Activating proteases are either the cysteine proteases caspase-1, -3, -4, -5, or -8, or the serine proteases granzyme (Gzm) A and B, neutrophil elastase and cathepsin G (**Table 2**). For example, GSDMD is activated by caspase-1 upon inflammasome activation or caspase-4/-5 (caspase-11 in mice) upon LPS-induced activation (63, 64). Then, pores of NT-GSDMD formed in the plasma membrane allow the release of pro-inflammatory cytokines and induce pyroptosis. However, when cells undergo apoptosis, GSDMD is cleaved by caspase-3 at aspartate 87 in NT-GSDMD, causing its inactivation (100, 104). In contrast, caspase-3 activates GSDME inducing secondary necrosis via NT-GSDME pores in the plasma membrane or apoptosis upon insertion in the mitochondrial membrane (101, 106). Interestingly, human GSDMA and murine GsdmA1 are activated by an exogenous pathogen-derived protease, and the induction of pyroptosis in keratinocytes prevents the spreading of the pathogen (80, 81). In contrast, GSDMB lacks autoinhibition, and the full-length protein already possesses bioactivity (85).

**Table 2:** GSDM activating and inactivating proteases.

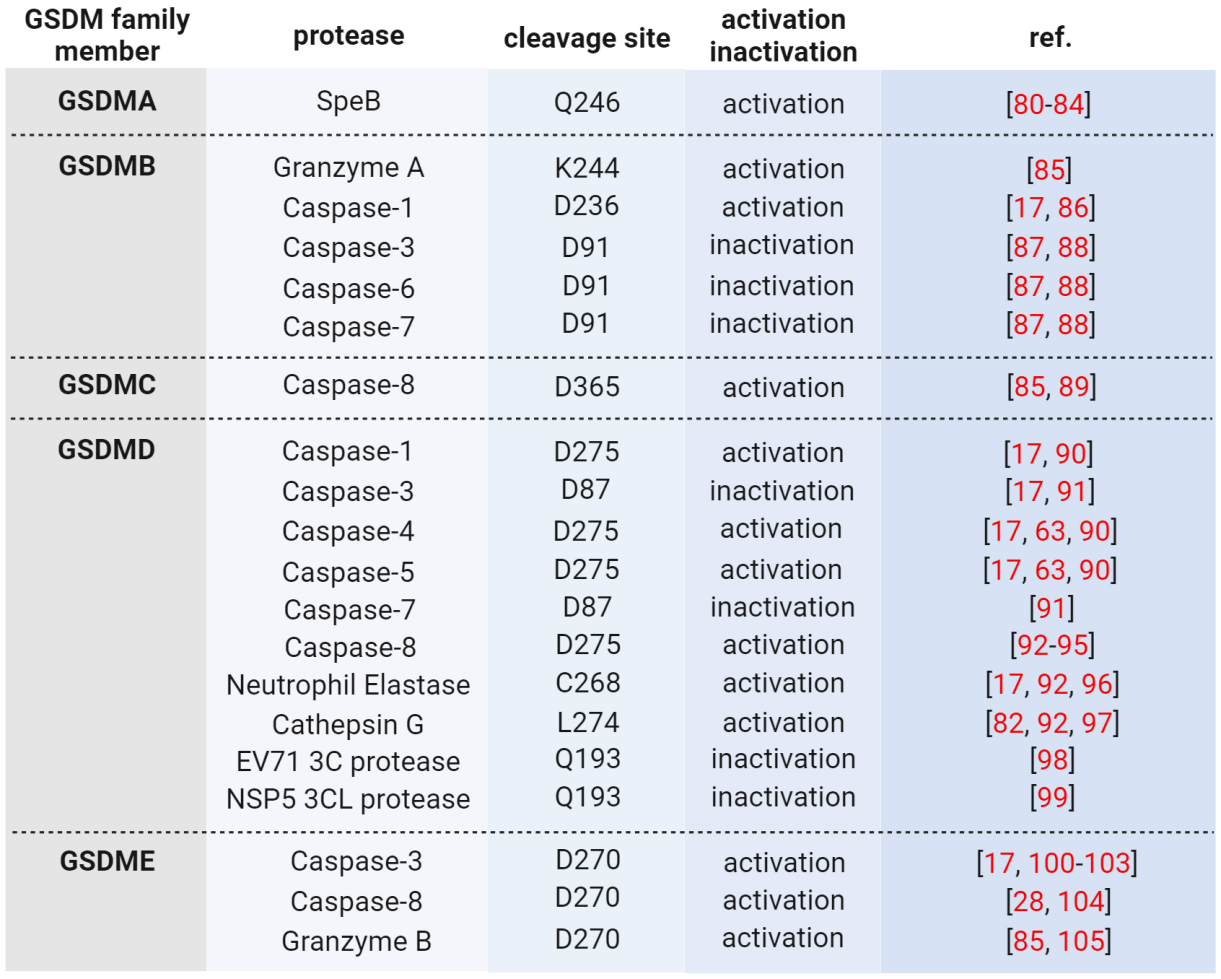

**Figure 1:**
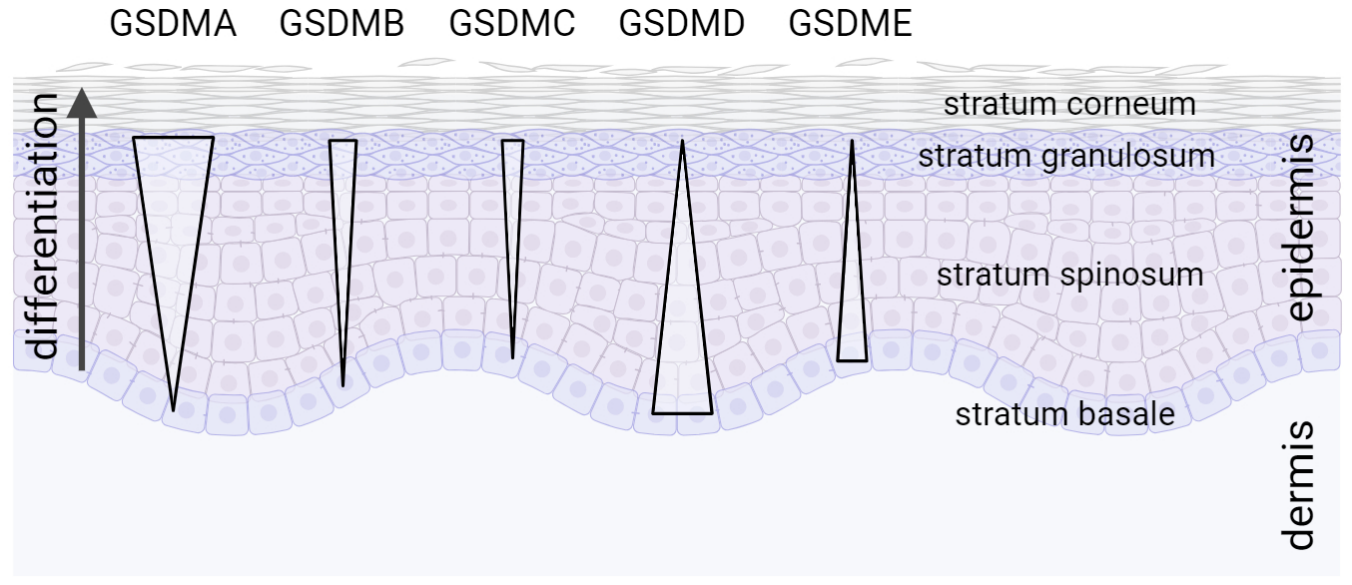
Expression gradient of GSDM family members in the epidermis. All GSDM family members are expressed in human skin in a differentiation-specific manner in stratified layers of the epidermis (108).

Gasdermins are widely expressed in different tissues and cells [see excellent reviews by (16, 17, 73, 107)]. However, the skin and keratinocytes seem to be the only tissue and cell type, respectively, expressing all GSDM family members (108). Whereas GSDMC, GSDMB, and particularly GSDMA expression is induced upon keratinocyte differentiation, levels of GSDMD and GSDME are lower in differentiated keratinocytes compared to proliferating cells (**Figure 1**). This expression pattern suggests important and differentiation-specific roles of GSDMs in human skin. It raises questions about the molecular mechanisms underlying their activation and the (patho)physiological functions of GSDMs in the skin.

## Auto-inhibition and activation

**Figure 2:**
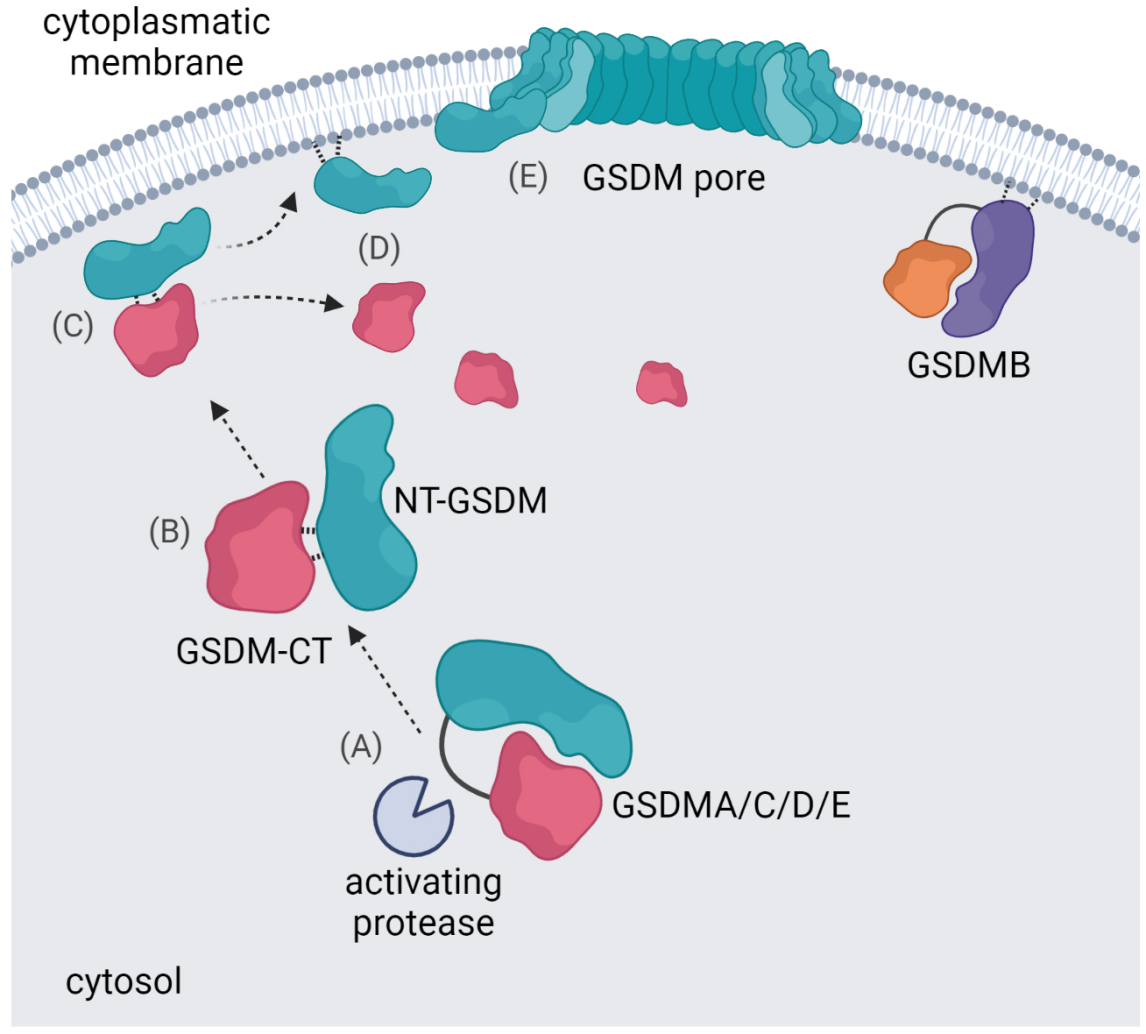
Gasdermin pore formation. An activating protease cleaves the gasdermin protein in the linker region between NT-GSDM and GSDM-CT **(A)** the NT-GSDM and GSDM-CT subunits remain associated in a complex **(B)** the NT-GSDM/GSDM-CT complex interacts with a membrane **(C)** and dissociates upon this interaction **(D)** NT-GSDM subunits oligomerize and form pores **(E)** GSDMB has a unique ability to interact with the cytoplasmatic membrane in its full-length form.

Apart from GSDMB, all GSDM family members are expressed in an auto-inhibited state (17). NT-GSDM is the effector fragment as ectopic expression of NT-GSDMA/B/C/D or -E induces pyroptosis (65, 101). This demonstrates that GSDM-CT inhibits NT-GSDM, and it is believed that the pore-forming activity of GSDM family members, except for GSDMB, is inhibited by similar molecular mechanisms (109). After proteolytic processing, NT-GSDM and GSDM-CT remain associated in a complex (92, 109). However, cleavage unveils the ability of GSDMs to bind liposomes with negatively charged phospholipids allowing them to interact with the inner leaflet of the plasma membrane (66). The crystal structure of GSDMD or GsdmA3 pores revealed conserved basic amino acids essential for binding acidic lipids and a hydrophobic part for membrane interaction (110). The NT-GSDM/GSDM-CT complex dissociates upon interaction with a membrane, and NT-GSDM forms oligomers and pores (**Figure 2**) (92, 109). The structure of GsdmA3 revealed two hydrophobic interdomain interfaces in NT-GsdmA3 and GsdmA3-CT, which are highly conserved in the gasdermin family (65, 78, 111). Indeed, mutating hydrophobic amino acids in this region activates full-length GSDMA, C, D, and E, causing cytotoxicity (16, 65). In GSDMB-CT, two α-helices, conserved in other GSDMs and required for interaction with the NT-fragment, are missing (87, 111). As a result, already full-length GSDMB binds to membranes (in contrast to other wild type GSDMs), although the pore-forming activity is still blocked (85).

Wild type GSDMs are activated - and in part also inactivated - by proteolytic processing through different proteases, particularly caspases (**Table 2**). For example, GSDMD is activated by caspase-1 at Asp275 in the linker between NT-GSDMD and GSDMD-CT upon canonical inflammasome activation (64) and by caspase-4/-5/-11 upon noncanonical inflammasome activation (63, 73). Furthermore, triggered by extrinsic apoptosis, caspase-8 can process GSDMD (17, 28, 93, 94) as well as the serine proteases neutrophil elastase (96, 112) and cathepsin G (97). In contrast, caspase-3, when activated during apoptosis, inactivates GSDMD by cleavage at Asp87 in its pore-forming aminoterminal domain (91). However, caspase-3 cleaves and activates GSDME at aspartate 270, and NT-GSDME pores accelerate slow apoptotic cell death to faster pyroptotic cell death (100, 101). GSDME supports anti-tumor immunity, and its expression is often suppressed in cancer (105). Furthermore, GSDME in cancer cells is also activated by NK and cytotoxic T cells upon delivery of granzyme B through perforin pores (109). Interestingly, the crystal structures of caspase-1/-4/-11 with GSDMD-CT revealed an interaction of the protease with the latter at a substrate-binding exosite remote from the active site (90, 113).

**Figure 3:**
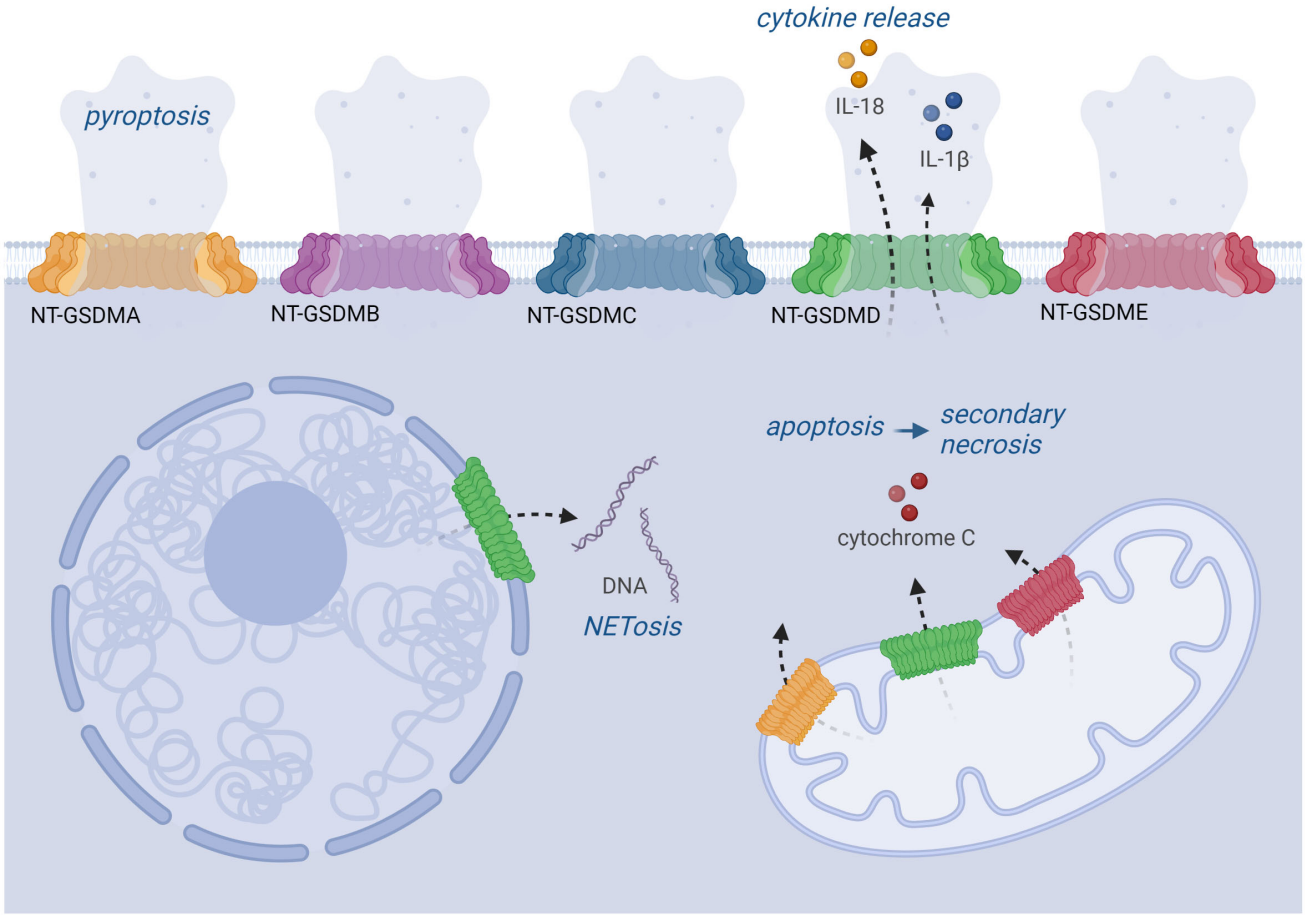
Localization and function of GSDM pores. Upon activation by protease cleavage, GSDMs can be localized in various cell membranes. All NT-GSDMs can target the plasma membrane where they cause its permeabilization leading to pyroptosis. GSDMD pores also enable the secretion of IL-1β and IL-18 cytokines. GSDMA, D, E can also be implemented into the mitochondrial membrane, where they cause the release of cytochrome C triggering the apoptotic pathway. Moreover, GSDMD can also target the nuclear membrane, which plays a role during the NETosis of neutrophils.

NT-GSDMD is also detected extracellularly but cannot insert from outside into membranes of adjacent cells to induce pyroptosis (66, 73). NT-GSDMs bind to different types of membranes. Although GSDMD induces cytokine release and pyroptosis in most cell types due to pore formation in the plasma membrane, it binds to the nuclear membrane in neutrophils, thereby inducing NETosis (**Figure 3**) (112, 114). Neutrophils express lower levels of ASC and caspase-1, and this lower amount of ASC specks might result in lower levels of active caspase-1 upon inflammasome activation and localization of GSDMD pores only in the nuclear membrane (114–116). NT-GSDMD and –E also insert into the mitochondrial membrane causing its permeabilization and release of ROS and cytochrome C; the latter activates caspase-9 leading to caspase-3 activation and apoptosis (**Figure 3**) (106). This is most likely mediated by binding to the negatively charged phospholipid cardiolipin in the mitochondrial membrane. Moreover, cardiolipin is also part of bacterial membranes, and indeed gasdermins can lyse protoplasts of *Bacillus megaterium* and *E. coli* (66, 109).

Characterization of the structure of GsdmA3 and GSDMD pores demonstrates that GSDMs represent a novel class of pore-forming proteins (65, 78). GSDM pores are structurally different from pore-forming perforin, BAX/BAK pores, which are formed during intrinsic apoptosis, or MLKL (mixed lineage kinase domain-like) pores induced by phosphorylation through RIP3 (receptor interacting serine/threonine kinase 3) in necroptosis (117). GsdmA3 pores are characterized by an inner diameter of 180 Å with a 27-fold symmetry, and GSDMD pores by a 215 Å diameter and a 33-fold symmetry (78, 110, 111). This size is sufficient for releasing the small IL-1 family members but not for larger proteins such as LDH (lactate dehydrogenase), which is a stable tetramer of 140 kDa. LDH is frequently used to quantify lysis and pyroptosis of cells due to its cytoplasmic localization and extracellular release when the cells are damaged. Recent studies suggest that GSDMD pores can quickly progress to sizes of 8000 Å and thereby accelerate pyroptosis (92, 118). Interestingly, the GSDMD pore is negatively charged and favours releasing neutral and positively charged proteins such as mature IL-1β over acidic proIL-1β (110).

## Pyroptosis and hyperactivation

Pyroptosis and NETosis are defined as gasdermin-induced necrotic-like types of regulated cell death, which support inflammation (17, 18, 112, 114). In necroptosis, caspases are not required for this type of cell death. In contrast, at least initially, apoptosis is a caspase-dependent non-lytic type of regulated cell death, which is immunologically silent. When apoptotic cells are not taken up by other cells upon exposing eat-me signals, they can undergo secondary necrosis mediated by caspase-3-dependent GSDME activation (100, 119). However, it should be considered that secondary necrosis does not resemble pyroptosis because activation of the apoptotic machinery results in the degradation of pro-inflammatory molecules and proteins. Pyroptosis has at least three consequences; it allows the release of pro-inflammatory cytokines, such as IL-1β, and IL-18, eliminates the replicative niche of pathogens upon infection and causes cell death (120, 121). Pyroptosis is characterized by the ballooning of the dying cell due to the osmotic uptake of water (18, 122). Interestingly, it has been suggested that NINJ1 (ninjurin-1), a transmembrane protein, is required for plasma membrane rupture in murine bone marrow macrophages but not for releasing IL-1β and IL-18 (123). Furthermore, in these cells, NINJ1 is also required for toxin-, secondary necrosis- and necroptosis-associated lysis (the latter only partially) (124). NINJ1 also plays a central role in plasma membrane rupture in other cell types (125). However, neutrophils do not undergo pyroptosis, presumably because they express low levels of ASC and caspase-1 (120). Furthermore, human keratinocytes do not undergo massive pyroptosis upon inflammasome activation, although they secrete high levels of IL-1β (48, 126). GSDMD-dependent pore formation and cytokine secretion from living cells that do not undergo pyoptosis is also termed hyperactivation and is a common phenomenon (127–132).

GSDM and MLKL pore formation is antagonized by membrane repair mechanisms regulated by the endosomal sorting complexes required for transport (ESCRT)-III machinery (16, 17). Upon the formation of GSDM pores, Ca^2+^ enters the cell and activates ESCRT-III. Then, the damaged plasma membrane is repaired upon budding and shedding of vesicles (133). Interestingly, these exosomes often contain IL-1β and, consequently, besides the release through pores and pyroptosis, the ESCRT-dependent repair of GSDM pores represents a third mechanism of cytokine release (17).

## GSDMA

Humans express a single GSDMA protein, while mice express GsdmA1, GsdmA2, and GsdmA3 (13). GsdmA1 was first discovered in 2000 and is expressed by epithelial cells in the upper gastrointestinal tract and skin (10), macrophages, and T cells (134, 135). Proliferating human keratinocytes and the SCC cell line A431 express GSDMA protein (80, 81), but its mRNA expression is strongly induced upon keratinocyte differentiation, suggesting a particularly important role in the outer epidermal layers (108). This was confirmed at the protein level since GSDMA/GsdmA is expressed in the upper epidermis and the inner root sheath and hair shaft by differentiated cells as well as by mature sebocytes (136). The expression of GSDMA is suppressed in esophageal, gastric, and skin cancer, suggesting a role as a tumor suppressor (107, 136, 137). SNPs of *GSDMA* are associated with systemic sclerosis (SSc), inflammatory bowel disease (IBD), and childhood asthma (138–141). Expression of GSDMA is induced in monocyte-derived macrophages of patients suffering from SSc (139).

In mice, at least 11 point mutations of GsdmA3 have been described, which cause alopecia (11, 17, 142). Although the underlying molecular mechanisms are only partially understood, it has been suggested that alopecia starting at day 22 of embryonic development is caused by inflammation and loss of bulge stem cells (142–144) and mediated by a role of GsdmA3 in apoptosis-driven catagen induction (145). Furthermore, these mutations cause thermogenesis in brown adipose tissue, a phenotype mediated by JNK activation and IL-6, which is rescued by immune suppression (146). Alopecia-causing mutations of GsdmA3 are gain-of-function mutations because mice lacking expression of GsdmA1/2/3 do not show a spontaneous phenotype (75, 81, 107, 144). Overexpression of GsdmA3 in HaCaT keratinocytes or HEK293 does not harm the cells (74), but overexpression of NT-GsdmA3 in the latter cell type induces lytic cell death (107), and overexpression of GsdmA3 in murine epidermis results in hyperplasia and inflammation (144). As mentioned above, GSDM-CT inhibits the pore-forming activity of NT-GSDM, and the alopecia-inducing mutations of GsdmA3 are located in GsdmA3-CT, disrupting autoinhibition (74). The crystal structure of GsdmA3 confirmed the inhibition of NT-GsdmA3 by a hydrophobic stretch of amino acids in GsdmA3-CT. Alopecia-inducing mutations weaken this interaction and cause spontaneous pore formation (65). Cryo-EM demonstrated massive structural changes upon lipid binding of GsdmA3 induced by alopecia-inducing mutations or upon proteolytic processing (17, 78).

Although overexpression of NT-GSDMA induces pyroptosis/necrosis via pore-formation in the plasma membrane (17, 65), GsdmA3/GSDMA also targets the mitochondrial membrane inducing ROS formation and disruption of mitochondria (74, 82, 83). Compared to NT-GSDMD, NT-GSDMA binds weaker to phosphoinositides located in the plasma membrane but stronger to cardiolipin, which is associated with the mitochondrial membrane (65, 66, 82). NT-GsdmA3 associates with Hsp90, and this complex is delivered to Tom70, a mitochondrial import receptor, and subsequently interacts with the mitochondrial chaperone Trap1 (83). As a consequence, mitochondria increase ROS production and lose their membrane potential. Furthermore, GSDMA/GsdmA3 can also lyse protoplasts of *Bacillus megaterium* (73, 109).

Until recently, neither a biological function of GSDMA nor an activating protease was known (84). Interestingly, two groups have shown that in keratinocytes, GSDMA is cleaved and activated by a pathogen-derived protease called SpeB, a virulence factor of the Gram-positive Group A *Streptococcus* (GAS) (80, 81). SpeB cleaves and activates GSDMA upon overexpression of SpeB or infection by GAS. Thereby GSDMA acts as a sensor for the pathogen. Moreover, cleavage at Gln246 induces GSDMA activation and pyroptosis, demonstrating that GSDMA also acts as an effector (80, 84). In mice, SpeB cleaves and activates GsdmA1. Most importantly, GsdmA1 knockout mice suffer from less skin inflammation upon infection with GAS. However, this is associated with uncontrolled systemic bacterial dissemination and an increased death rate of infected mice, demonstrating that GsdmA1 expressed by keratinocytes acts as a guardian protein in the skin (80, 81). Interestingly mice lacking expression of GsdmA1 and GsdmA3 in the epidermis have a defect in skin barrier repair upon repeated epidermal barrier disruption (147). These findings suggest a role of GsdmAs in keratinocyte differentiation.

## GSDMB

GSDMB does not have a rodent ortholog (13) and possesses unique properties within the GSDM family, only recently summarized in an excellent review (85). Compared to GSDMA, GSDMB is much broader expressed, mainly by epithelial cells in the airway, gastrointestinal tract, or liver, but also by immune cells (73, 148–150). In the skin, expression of GSDMB is induced upon differentiation (108). From the *GSDMB* gene, six different splice variants are expressed, but their expression patterns and properties are poorly characterized (85).

SNPs of GSDMB are associated with several chronic inflammatory diseases, such as IBD (138, 151), rheumatoid arthritis (152, 153), and asthma (154, 155). Isoform 1 of GSDMB is overexpressed in bronchial epithelial cells of asthma patients and is located in the nucleus (156). GSDMB expression is suppressed in skin lesions of patients suffering from psoriasis vulgaris, suggesting a role in the pathogenesis of this inflammatory skin disease (157). Most importantly, GSDMB expression is increased in different types of cancer, including breast, cervical, hepatic, and gastrointestinal cancer (149, 158–160), and its high mRNA expression is associated with poor prognosis in breast cancer (161). Therefore, GSDMB might be an oncogene (21).

Although overexpression of NT-GSDMB induces necrosis (65), already full-length GSDMB binds to membrane lipids (87). The lack of two α-helices in GSDMB-CT, responsible for the binding of NT-GSDM, results in the direct binding of full-length GSDMB to membranes (85, 87, 111). Most importantly, GSDMB supports proliferation and motility, which is particularly important for cancer cells (162–164). However, the underlying molecular mechanisms are poorly understood (85). Different proteases cleave GSDMB. Apoptotic executioner caspases cleave in NT-GSDMB, e.g., caspase-3 at Asp91, causing protein inactivation (87). In contrast, granzyme A delivered through perforin pores and expressed by cytotoxic T lymphocytes and natural killer cells, activates GSDMB by cleavage in the linker region, inducing pyroptosis in epithelial cells (165). Activation of GSDMB at Asp236 by caspase-1 causes pyroptosis (86). Conversely, GSDMB enhances caspase-4 activity and promotes noncanonical pyroptosis (88). However, it should also be mentioned that the pore-forming activity of GSDMB or NT-GSDMB is controversial because GSDMB might neither interact with phospholipids in the plasma membranes nor form oligomeric pores (85, 166). In summary, GSDMB is clearly different from the other family members and possesses biological activities beyond cell death without a need for proteolytic activation (85).

## GSDMC

In contrast to humans, which express a single GSDMC protein, mice express four different GsdmC orthologs. However, the roles of these orthologs are poorly characterized (13). GSDMC is expressed in epithelia, such as the esophagus, small intestine, and colon, but it lacks in immune cells (134, 135, 148, 167). In human keratinocytes, GSDMC expression is slightly induced upon differentiation (108) and UVB radiation (168). UVB-induced GSDMC expression in HaCaT keratinocytes is Ca^2+^-dependent and regulated by TRPV1 (transient receptor potential cation channel subfamily V) (168), which is required for ERK/JNK activation and MMP1-induction (107, 169). The role of GSDMC in cancer development is a matter of debate (17). In metastatic melanoma (167) and colorectal cancer, GSDMC is considered an oncogene (73), as its expression is increased and supports cancer cell proliferation (170). In contrast, in esophageal squamous carcinomas, GSDMC expression is suppressed (137, 148). Expression of GSDMC is induced in lumbar disc degeneration, and the condition is associated with SNPs of *GSDMC* (171, 172). Furthermore, it has been shown that PD-L1 and p-Stat3 induce GSDMC expression synergistically, and caspase-8 activated upon TNFα stimulation cleaves GSDMC at Asp365, inducing pyroptosis of cancer cells (89).

## GSDMD

In contrast to other GSDM family members, GSDMD is not only expressed by epithelial and immune cells but more ubiquitously (134, 135). It is the best-characterized GSDM, highlighted in recently published excellent reviews (17, 73, 120). GSDMD is a downstream target of all inflammasome complexes and, therefore, plays an important role in inflammation (34, 173). Caspase-1, which is activated upon the assembly of inflammasome complexes, is the main activating protease of GSDMD (69). Still, GSDMD is also cleaved by other inflammatory caspases upon non-canonical inflammasome activation and by caspase-8, cathepsin G, and neutrophil elastase. However, the physiological significance of these cleavage events is partially unclear (17, 63, 69, 73). The *Yersinia* effector protein YopJ inhibits the protein kinase TAK1 and NF-κB (95). Under these conditions, TLR4 or TNFR activation results in the activation of caspase-8 induced by RIPK1 and the Rag-Ragulator complex. Then, caspase-8 activates GSDMD inducing pyroptosis, which is required to counteract *Yersinia* infection (95). In contrast, caspase-3 and caspase-7 inactivate GSDMD by cleavage at Asp87 and thereby prevent pyroptosis (91, 92). Interestingly, in some pathogens, mechanisms evolved, causing the inactivation of GSDMD and prevention of pyroptosis and cell death, thereby allowing pathogen replication. GSDMD is inactivated by the cleavage at Gln193 by the viral 3C protease of enterovirus 71 (98) as well as by the coronavirus 3CL protease (99). However, in both cases, caspase-3-induced GSDME activation rescues cell death (99, 102).

NT-GSDMD binds to phosphatidylinositol phosphate and phosphatidylserine located at the inner leaflet of the plasma membrane (66). This ensures that pyroptosis is not induced by GSDMD activation in neighboring cells either by ESCRT-dependent repair of the plasma membrane and shedding of GSDMD pores or directly by cell lysis. In neutrophils, NT-GSDMD interacts with the nuclear membrane and induces NETosis (112, 114). Furthermore, NT-GSDMD binds to cardiolipin, which is situated in bacterial membranes and is able to kill bacteria (66, 107). However, cardiolipin is also a mitochondrial membrane component, and NT-GSDMD binding causes mitochondrial damage, ROS release, and cell death (82, 106).

Expression of GSDMD is regulated by IRF2, a transcription factor known to repress interferons (174). Most SNPs of *GSDMD* do not alter pore formation, and so far, none has been linked to disease (73). Mice lacking GsdmD expression do not show a spontaneous phenotype (175). Depending on the pathogen, knockout mice are either more or less susceptible to infection compared to control animals, and IL-1β release is rather delayed than inhibited (73, 176). Nevertheless, GSDMD plays essential roles in different models of inflammation (73). NOMID (neonatal-onset multisystem inflammatory disease) patients suffer from a severe auto-inflammatory disease caused by activating mutations of the *NLRP3* gene (35), and knock-in mice with a NOMID mutation show a similar phenotype (177). In these mice, additional ablation of GSDMD expression prevented all NOMID-associated inflammatory symptoms (178). Furthermore, in a mouse model for skin infection by *Staphylococcus aureus*, GsdmD expression contributes to host defense (179). In a mouse model for systemic lupus erythematosus, GsdmD exerts a protective role (180). Serum levels of patients suffering from adult-onset Still`s disease or systemic juvenile idiopathic arthritis, two auto-inflammatory diseases, are characterized by increased levels of IL-18 and NT-GSDMD suggesting important roles of the pore-forming protein in the pathology of these diseases (181). GSDMD might also contribute to scleroderma, a fibrotic skin disease (182). In non-small cell lung cancer, GSDMD expression is upregulated, supports metastasis, and is associated with a poor prognosis (183) and in general, GSDMD is differentially expressed in cancer (184).

In human keratinocytes, GSDMD expression is decreased upon differentiation (108). GSDMD clearly supports the release of IL-1β and -18 upon NLRP1 inflammasome activation in human keratinocytes without inducing pyroptosis (44, 48). NLRP1 activation with IL-1β and -18 release represents an early event induced by UVB radiation (45, 46, 185) and is followed by apoptosis that occurs significantly later and is not dependent on NLRP1 activation (185). Interestingly, UVB-induced IL-1β release is only delayed in GSDMD knockdown keratinocytes suggesting that other family members might compensate for this process (126). However, these cells undergo apoptosis earlier, demonstrating that in keratinocytes, GSDMD pores support survival, presumably due to the release of active caspase-1 and other caspases that induce apoptosis when they remain intracellularly (126, 185).

## GSDME

*GSDME* is the most ancient gene of the GSDM family and is ubiquitously expressed (15, 134, 135). Nevertheless, mutations of *GSDME* resulting in the expression of a truncated cytotoxic protein cause nonsyndromic hearing loss in humans, whereas phenotypes in other tissues are not described (14). It is not understood why these mutations cause only damage and a phenotype in the inner ear and not in other tissues and cells with high GSDME expression (17). *GSDME* expression is silenced by promoter methylation in different types of cancer (73, 186–190). Furthermore, *TP53* - a tumor suppressor gene that is frequently inactivated in many cancers – induces transcription of *GSDME* in cells with DNA damage (73, 191). Therefore, GSDME is a clear tumor suppressor (17, 73). The apoptotic executioner caspase-3 is the most important proteolytic activator of GSDME (101). Upon activation, NT-GSDME inserts into the plasma membrane inducing secondary necrosis. This pathway is induced by chemotherapeutic drugs in tumor cells (100). On the one hand, this directly accelerates cell death. On the other hand, GSDME activation might transform non-inflammatory apoptosis into inflammatory pyroptosis, activating anti-tumor immunity. Furthermore, NT-GSDME also binds to the mitochondrial membrane causing its damage upon pore formation and activating the mitochondrial pathway of apoptosis (106). NK and CD8^+^ cytotoxic T cells can deliver GzmB to tumor cells, which induces apoptosis upon activation of caspase-3 and downstream activation of GSDME, which causes pyroptosis. On top of that, GzmB can also directly cleave and activate GSDME itself. Described processes further support the anti-tumor function of GSDME (105, 192). Tumor cell death caused by GSDME activation can also be induced by chimeric antigen receptor T (CAR-T) cells (193). Treating melanoma patients with BRAF/MEK inhibitors induces GSDME-dependent pyroptosis and supports anti-tumor immune responses (192, 194). GSDME can compensate for the inactivation of GSDMD upon infection with coronavirus in epithelial cells or enterovirus 71 in Hela cells and induce pyroptosis (99, 102).

Expression of GSDME is decreased upon induction of differentiation of human keratinocytes (108). UVB irradiation of HaCaT keratinocytes induces GSDME activation and pyroptosis (195). Ablation of GsdmE expression in mice revealed that the pore-forming protein supports inflammation via the recruitment and activation of neutrophils into UVB-irradiated skin (196). UVB-induced GSDME-dependent pyroptosis of keratinocytes is antagonized by microRNA miR-133a-3p, but the underlying molecular mechanisms are incompletely characterized (197). Interestingly, infection of human keratinocytes with viruses, such as VSV or HSV-1, induces GSDME-dependent pyroptosis activated by caspase-3 and release of the alarmin IL-1α (103). Blocking this pathway enhances virus replication, demonstrating a protective function of GSDME in human keratinocytes and an organoid model of human skin (103).

## Conclusions and outlook

First, after their discovery in keratinocytes of the skin, GSDM family members lived mainly in the shadow. This changed dramatically with the identification of GSDMD as a substrate of inflammatory caspases and an effector protein of inflammasomes inducing pyroptosis in immune cells (63, 64). Today, GSDMs are well established as a novel family of self-inhibited pore-forming proteins activated upon proteolytic processing or by mutations. Due to its role as an inflammasome effector protein, GSDMD is still considered the most important family member, and its inhibition might represent a novel therapeutic approach for treating inflammatory diseases with the involvement of inflammasomes. Indeed, different molecules were discovered that inhibit GSDMD pore formation, such as disulfiram, fumarates, or necrosulfonamide (198–200). However, it is known that in most cases, inflammasome-induced IL-1β secretion is just delayed by GSDMD inhibition or knockout, most likely due to redundancy in the GSDM family (73). Therefore, a task for the future will be to analyze the crosstalk between GSDM family members. Here, epithelia, including the epidermis, are particularly challenging because all family members are expressed by keratinocytes in human skin (108), which suggests particularly important roles in the skin. However, our knowledge about the roles of GSDM family members in the skin is very limited, reflected by the low number of publications dealing with GSDMs in keratinocytes and the skin, particularly in humans. The varying number of members of the GSDM family in humans and mice indicates that the proteins are poorly conserved, limiting the relevance of experiments based on the murine model for humans. Interestingly, particularly GSDME seems to act as a tumor suppressor (73). Therefore, in contrast to GSDMD, which supports inflammation and inflammatory diseases, cancer treatment strategies aiming for the activation of GSDMs might be promising. Furthermore, it can be anticipated that more lysis- and pyroptosis-independent roles of GSDMs will be identified in the future. Even GSDMD activation does not necessarily result in pyroptosis. There are several examples of hyperactivated cells which release pro-inflammatory cytokines lacking a signal peptide via GSDMD pores without undergoing pyroptosis, such as human keratinocytes (44). In these epithelial cells, GSDMD activation even has a cytoprotective effect acting like a valve upon inflammasome activation since GSDMD pores also allow the release of activated cytotoxic caspases (126, 185). In the coming years, a significant challenge lies in identifying particular physiological stimuli responsible for triggering the activation of GSDMs. This activation is closely linked to their involvement in pathological and stress-related conditions and their potential contributions to maintaining homeostasis within the body, particularly on its surface in keratinocytes of the epidermis.

GSDMs exemplify an intriguing and distinctive protein family, and our knowledge regarding these pore-forming entities has expanded considerably since their initial identification. Nonetheless, it is plausible to consider that our current understanding merely scratches the surface, implying that there is much more to uncover and explore regarding GSDMs, opening up exciting opportunities for new insights and discoveries not only but also in the skin.

## Author contributions

MS: Visualization, Writing – original draft, Writing – review & editing. TK: Writing – review & editing. MDF: Writing – review & editing. PH: Writing – review & editing. HB: Funding acquisition, Project administration, Supervision, Writing – original draft, Writing – review & editing.
